# How food regulations help or hinder the implementation of policies to promote healthy population diets: a review of food regulations in the Western Pacific Region

**DOI:** 10.1017/S1368980025101687

**Published:** 2025-12-26

**Authors:** Katie Fries, Kathryn Backholer, Alexandra Jones, Fiona Sing, Erica Reeve

**Affiliations:** 1 Deakin University, Burwood, Australia; 2 The George Institute for Global Health, Sydney, Australia; 3 University of Auckland, Auckland, New Zealand

**Keywords:** Nutrition policy, Non-communicable disease prevention, Public health, Population health

## Abstract

**Objective:**

Governments are seeking to regulate food environments to promote health by restricting sales and marketing of processed foods high in fat, sugar and sodium. We aimed to evaluate whether the legal instruments in member states of the Western Pacific Region (WPR) mandate the declaration of nutrient composition for nutrients of concern in relation to Codex Alimentarius and non-communicable disease (NCD) prevention.

**Design:**

We undertook content analysis of legal instruments governing food quality and safety, documenting mandates for nutrient declarations in the WPR. Legal instruments were purposefully sourced through a systematic search of regional legal databases and Google. We performed qualitative and quantitative analysis, using an adapted version of Reeve and Magnusson’s Framework for Analysing and Improving the Performance of Regulatory Instruments.

**Setting:**

Legal instruments governing food quality and safety in twenty-eight member states of the WPR.

**Results:**

There was substantial variation in the nutrient declaration mandates within legal instruments, with only three out of twenty-eight countries mandating nutrient declarations in full alignment with Codex recommendations (energy, protein, available carbohydrate, fat, saturated fat, sodium and total sugars). Just four countries mandated the display of sodium, sugar, saturated fat and trans-fats, in line with NCD prevention recommendations. Sodium labelling was mandated in ten countries, sugar in seven and saturated fat in six.

**Conclusions:**

There is scope for countries to strengthen legal instruments for nutrient declarations to better support diet-related NCD prevention efforts. Regional support agencies can play a key role in promoting greater policy coherence and alignment with international best practice.

Non-communicable diseases (NCD) are the leading cause of death worldwide, responsible for 41 million (74 %) deaths in 2022^(1,2)^. NCD have a significant impact on sustainable development, and their reduction is recognised by the UN as a Sustainable Development Goal^(1)^. A key driver of NCD is the global shift towards diets high in processed foods, containing excessive amounts of sodium, free sugars and saturated and trans-fats^(2–4)^, alongside the transition to unhealthy food environments – the places where people are exposed to the food system and purchase, prepare and eat food^(2–4)^. Globally, these food environments are increasingly dominated by the promotion and sales of unhealthy foods, driving up consumption^(4,5)^.

The WHO has recently updated population dietary guidelines, providing clear evidence on the need to adopt food-related policies that reduce overall *demand* of processed foods that are high in saturated fats, trans-fats, free sugars and sodium (HFSS) and whose consumption is associated with negative health outcomes^(6)^. Policies that promote healthy food environments and restrict sales and consumption of HFSS foods are one of the most effective ways to alter systemic drivers of unhealthy population diets^(4)^ in order to reduce the incidence of diet-related NCD^(3)^ and improve the equity and health of populations^(7)^. Key policy recommendations by WHO include restricting unhealthy food and beverage marketing, incentivising food reformulation, comprehensive nutrition labelling policies including front-of-pack nutrition labelling, improving the healthiness of publicly procured foods and using fiscal policy to promote healthier diets^(6,8)^. Governments around the world have committed to various international agreements to work towards adopting these policies^(9)^.

One of the key ways to identify ‘healthy’ and ‘unhealthy’ foods for the purposes of these policies and regulations is through nutrient profiling, which is the science of categorising foods according to their nutritional composition for reasons related to preventing disease and promoting health^(10)^. Countries around the world have worked to develop nutrient profiling models that draw on dietary guidelines to inform thresholds that determine whether foods are considered ‘healthy’ and ‘unhealthy’ and should therefore be targeted by policy intervention^(7,11,12)^. WHO has also developed a series of regional nutrient profiling models that can be adopted or adapted. These vary but most commonly target foods based upon their saturated fat, total sugars (or added sugar) and/or sodium thresholds^(13,14)^. Countries have also developed their own nutrient profiling models for specific policy uses. For example, Australia uses a nutrient profiling model to underpin legislation for health and nutrition claims, as well as a voluntary Health Star Rating front-of-pack nutrition labelling system that includes energy, saturated fat, total sugars, sodium, protein and fruit, vegetable, nut and legume content^(15)^. Samoa incorporated a nutrient profiling model into its food safety and quality regulations in 2017, setting upper limits for the amounts of fat, saturated fat, sugar and sodium in food products. This was designed to support the future implementation of food environment regulations, such as food taxation^(16)^. The practical application of nutrient profiling models relies upon the availability of product-specific food composition information, primarily from nutrient declarations provided on the back-of-pack (BOP) food labels^(17)^. While intended primarily to provide consumers with nutrition information at the point of purchase, these BOP nutrient declarations have an important foundational role in supporting the implementation of additional food policies.

International standards for what is contained in a BOP nutrient declaration are contained in the Codex Alimentarius Commission’s Guidelines on Nutrition Labelling (CXG 2–1985), which recommends that nutrient declarations for energy, protein, available carbohydrate, fat, saturated fat, sodium and total sugars should be mandatory on pre-packaged food (Box 1)^(18,19)^. In order to facilitate trade in food, many countries bring their policies into alignment with Codex standards. However, as each country ultimately determines its own national regulation, there are still variations in what is actually conveyed across jurisdictions.

Box 1. Extract from Codex Alimentarius guideline on nutrition labelling (CXG 2-1985)

**Application of nutrient declaration**

Nutrient declaration should be mandatory for all prepackaged foods for which nutrition or health claims, as defined in the Guidelines for Use of Nutrition and Health Claims (CXG 23-1997), are made

Nutrient declaration should be mandatory for all other prepackaged foods except where national circumstances would not support such declarations. Certain foods may be exempted for example, on the basis of nutritional or dietary insignificance or small packaging.

**Listing of nutrients** Where nutrient declaration is applied, the declaration of the following should be mandatory:

- Energy value; and
- The amounts of protein, available carbohydrate (i.e. dietary carbohydrate excluding dietary fibre), fat, saturated fat, sodium and total sugars; and
- The amount of any other nutrient for which a nutrition or health claim is made; and
- The amount of any other nutrient considered to be relevant for maintaining a good nutritional status, as required by national legislation or national dietary guidelines*
*Countries where the level of intake of trans-fatty acids is a public health concern should consider the declaration of trans-fatty acids in nutrition labelling.

Bringing national nutrient declarations into alignment with international Codex standards can facilitate the uptake of WHO’s recommended NCD prevention policies given their reliance on targeting nutrients such as sodium, sugars, trans-fat and saturated fat. The lack of uptake of mandatory and comprehensive BOP nutrient declarations has been noted as a barrier to the implementation and enforcement of diet-related NCD prevention policies in some member states^(20,21)^. For example, several countries have found it challenging to implement salt reduction strategies because sodium is not required by nutrient declarations in their countries, meaning the sodium content in products cannot be tracked over time^(20,22,23)^.

The Western Pacific Region (WPR) comprises thirty-eight countries and areas, with nearly 2·2 billion people living in the region^(24)^. Our aim was to evaluate whether the legal instruments in place across member states in the WPR mandate the declaration of nutrient composition for nutrients of concern in relation to Codex Alimentarius and for the quantification of nutrients of concern for NCD prevention. We chose to focus on the WPR because diet-related NCD are attributable to 86 % of mortality globally^(25)^, and governments in this region have committed to implementing a number of food environment policies to address them^(26)^.

## Methods

We undertook content analysis of legal instruments governing food quality and safety to document mandates for nutrient declarations in countries across the WPR. Content analysis provided a systematic method of coding and analysing the legal instruments against a framework for best-practice regulatory design for food environment policies^(27)^.

### Data source

We reviewed all relevant national legal instruments governing food quality and safety (depending on national government law frameworks)^(27)^. We sourced these using Google’s search engine and regional legislative databases, including the Pacific Islands Legal Information Institute website. We used search terms such as ‘food labelling regulations’ and ‘nutrition labelling guidelines’ and ‘food safety’^(28,29)^. For further information on our search strategy, see Appendix A.

### Data analysis

Our data extraction and analysis were informed by Reeve and Magnusson’s public health law framework^(30)^. This framework was developed for evaluating and improving the performance of regulatory instruments by presenting a set of ‘regulatory domains’ against which regulatory instruments can be assessed for strengths and weaknesses. We adapted the framework by omitting ‘regulatory form’ because we were only interested in legal instruments and not other policy instruments (e.g., voluntary guidelines) and by specifying the types of data to be extracted for each domain as it relates to BOP nutrient declarations (Table 1)^(31,32)^.

**Table 1.** Data extraction framework adapted from Reeve and Magnusson^(30)^

Regulatory domain	Best practice recommendation	Data extracted
General information		Country Name and type of legal instrument
Regulatory purpose	Mandated legal measures with clear, measurable objectives against which the success of regulation can be assessed	Legislative objectives
Regulatory substance	Key terms and conditions are clearly defined; regulatory rules are sufficiently expansive to achieve regulatory objectives; legal instruments mandate nutrient declarations in line with Codex Alimentarius. Specifically: The nutrient declaration should be mandatory for pre-packaged foods with limited exceptions (e.g., for nutritional insignificance or small packages). Where required, the nutrient declaration should include: Energy value: the amounts of protein, available carbohydrate (i.e., dietary carbohydrate excluding dietary fibre), fat, saturated fat, sodium and total sugars, with consideration of including trans-fats too, where this intake is of public health concern The amount of any other nutrient for which a nutrition or health claim is made The amount of any other nutrient considered to be relevant for maintaining a good nutritional status, as required by national legislation or national dietary guidelines	Definitions of key terms Standard nutrients to be declared and criteria for inclusion of additional nutrients (e.g., where an additional nutrient is the subject of a health and nutrition claim made elsewhere on pack).
Regulatory governance	Administration by an independent body, which monitors and enforces the scheme and arranges for external review of its performance. Monitoring indicators are provided^(30)^, and the administrative body should possess a range of sanctions, including negative publicity, fines and enforceable undertakings	Process and delegation for monitoring and enforcement Enforcement mechanism and penalties of non-compliance

In line with our data extraction framework (Table 1), we extracted data into an Excel form on regulatory purpose (legislative objectives), regulatory substance (definitions of key terms, nutrients mandated for display on BOP labels and criteria for display of nutrients where a health or nutrition claim regarding the nutrient is made on the item) and policy implementation (the processes for monitoring policy compliance and enforcement mechanisms and penalties for non-compliance). We generated quantitative summaries and then benchmarked the data against CODEX guidelines and the nutrients that are included in WHO’s recommended policies to promote healthier diets (Table 1)^(8,33)^.

## Results

We identified food-related legal instruments in twenty-eight of the thirty-seven countries in the WPR. Eight countries were protectorates of other states and therefore subject to external laws, and we were unable to find translations for one country (Macao). We found that the legal instruments used to guide food labelling included Acts (twenty-eight), Regulations (seventeen), Standards (three), Codes (two), Laws (two), a Decree (one) and an Order (one), totalling fifty-four documents. Legal instruments related to food labelling were coded collectively at the country level.

### Regulatory purpose

Reeve and Magnusson’s Framework states that legal measures should include measurable objectives against which the success of the law can be assessed^(30)^. Eleven countries included objectives specifically related to labelling (Australia, Cambodia, China, Japan, Laos, Mongolia, New Zealand, Northern Mariana Islands, Palau, the Philippines and the Republic of Korea). These objectives varied but were generally focused on four key areas (Table 2). These included human rights and health, including the prevention of food-borne illnesses and food safety (seventeen countries), provision of health information to consumers (ten countries), food production standards (nine countries) and trade and administration (nine countries). None of the legal instruments referred to NCD or the promotion of healthy diets or nutrition within the policy objectives.

**Table 2.** Major themes of policy objectives in food-related legal instruments of the Western Pacific Region

Policy objectives*	Number of countries	Examples
Human rights and health	17	Prevent food-borne illnesses Food safety
Health information for consumers	10	Provision of information to consumers to enable informed choices Protect consumers from deception
Food production and distribution standards	9	Prevention of misleading/deceptive conduct/fraud Promote smooth production and distribution of food
Trade and administration	9	Regulatory framework to facilitate efficiency for the food industry Promote fair/sound trade practices in relation to food and related matters

*Nine countries did not state any objectives.

### Regulatory substance is sufficiently expansive to achieve aims

Legal instruments should define key terms and mandate nutrient declarations in line with the Codex Alimentarius Guideline on Nutrition Labelling (CXG 2–1985), which states that where a nutrient declaration is applied, display of the following information should be mandatory: ‘energy value; the amounts of protein, available carbohydrate (i.e., dietary carbohydrate excluding dietary fibre), fat, saturated fat, sodium and total sugars; the amount of any other nutrient for which a nutrition or health claim is made; and the amount of any other nutrient considered to be relevant for maintaining a good nutritional status, as required by national legislation or national dietary guidelines’. The guideline also contains a footnote that countries where the level of intake of trans-fatty acids is a public health concern should consider the declaration of trans-fatty acids in nutrition labelling^(18)^.

We found that twenty-six countries included policy definitions in their legislation, though not all defined key terms related to nutrition labelling. Nutrients were defined by six countries, labels were defined by twenty-six countries, health or nutrient claims were defined by eleven countries and nutrition labelling was defined by seven countries.

We found that thirteen countries mandated nutrient declarations on all pre-packaged foods (with some limited exceptions), while five countries (Brunei, China, Cook Islands, Papua New Guinea and Singapore) mandated nutrient declarations only where a claim is made, and seven countries (Cambodia, Laos, Nauru, Northern Mariana Islands, Tuvalu, Vanuatu and Vietnam) did not specify a mandate for nutrient declarations. Legislation for the Cook Islands referred to an unavailable section of an Act for details on nutrition labelling. Two of the countries (Tonga and Mongolia) without a mandate deferred to the Codex Alimentarius General Standard for the Labelling of Pre-packaged Food. Three countries (Guam, Hong Kong, the Philippines) required the display of all the nutrients specifically named by the Codex Alimentarius, being energy, protein, carbohydrates, fat, saturated fat, sodium and total sugars.

Three countries (Guam, Hong Kong and the Philippines) gave the most comprehensive nutrient declaration mandates, requiring the display of energy value, protein, available or total carbohydrate, fat, saturated fat, sodium and total sugars. Of the countries mandating nutrient declarations, only four countries (Guam, Hong Kong, the Philippines and the Republic of Korea) mandated the display of all five of the group of nutrients recommended for display by WHO that are relevant to diet-related NCD prevention (sodium, sugar, saturated fat and trans-fat) (Table 3).

**Table 3.** Comparison of CODEX and WHO nutrient labelling requirements and what is mandated by countries in the Western Pacific Region

Nutrient required by Codex	Number of countries in which nutrient is mandatory
Energy value	11
Protein	13
Carbohydrate	13^*^
• of which was total	10
• of which was available	4
Fat	13
Saturated fat	6
Sodium	10
Sugars	7
• of which were total sugars	6
• of which were added sugars	1
Other nutrients	
Trans-fat	4
Fibre	2
Benchmarking	
Number of countries mandating the display of *all* nutrients required by CODEX (energy, protein, available/total carbohydrate, fat, saturated fat, sodium and total sugars)	3 (Guam, Hong Kong, the Philippines)
Number of countries mandating the display of nutrients recommended by WHO for NCD prevention (saturated fats, trans-fats, sugars and sodium)	4 (Guam, Hong Kong, the Philippines, the Republic of Korea)^**^

NCD, non-communicable disease.

* Hong Kong includes both total and available carbohydrates: total carbohydrates can be used instead of available carbohydrates if dietary fibre is also included; hence, thirteen countries require the display of carbohydrates.

** A number of countries mandated the display of all four nutrients recommended by WHO as relevant to diet-related NCD prevention but stipulated that one or more of these nutrients were only required to be declared in the instance where a claim was made (e.g., Australia, New Zealand and Malaysia)’.

**Figure 1:**
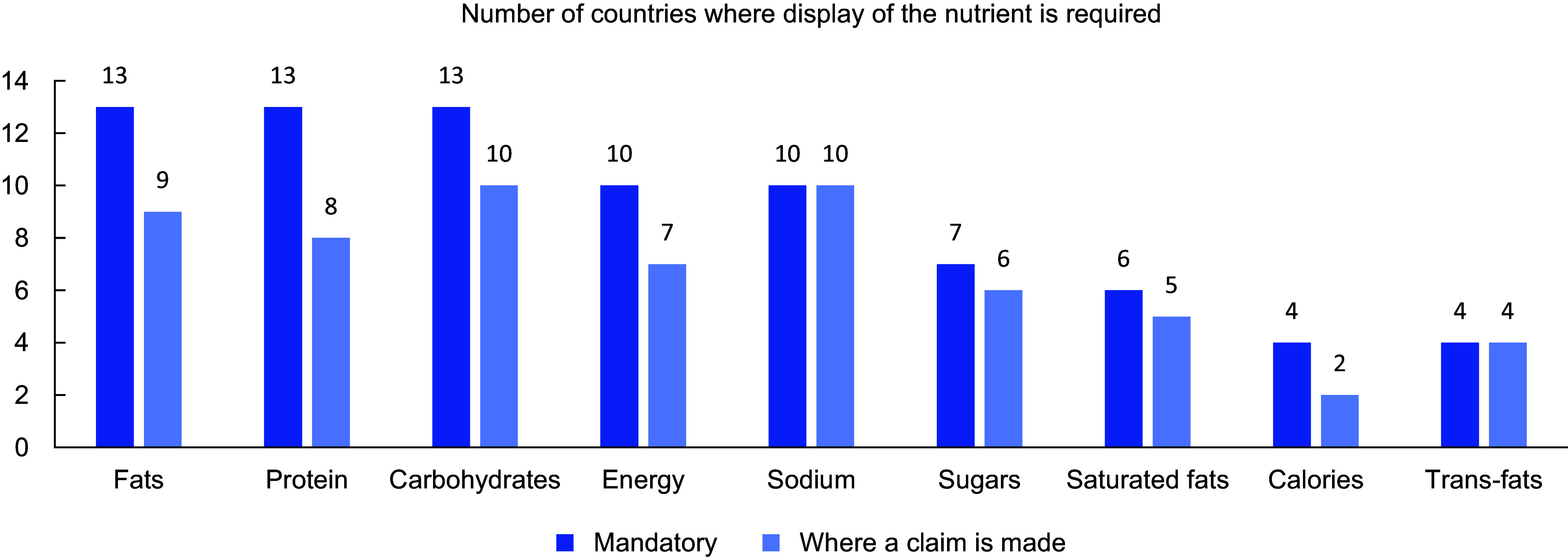
The number of countries requiring the display of specific nutrients, where the display is mandatory and, second, where a claim is made.

In total, thirteen countries mandated the display of information for total fat and protein (Table 3) (Fig. 1), thirteen countries mandated the display of carbohydrates, while eleven countries mandated the display of energy. Sugar was required for display by seven countries (total sugars in six countries, added sugar in one country). Sodium was required for display by ten countries. Saturated fat was required for display by six countries, while trans-fat was required for display by four countries. Fibre was mandated for display in two countries. The requirements of two countries (Cook Islands and Mongolia) were unknown as they referenced inaccessible documents.

We found that four countries (Brunei, Cook Islands, Papua New Guinea and Singapore) only mandated nutrient declarations when a health or nutrition claim was made on pack but did not require nutrient declarations otherwise. Some countries mandated the display of many of the nutrients recommended for display by WHO relevant to diet-related NCD prevention but stipulated that one or more of these nutrients were only required to be declared where a claim was made. For instance, in Australia and New Zealand, nutrient declarations are required for fats, sugars and sodium, but in the instance of trans and saturated fats, a declaration is only required if a claim is made against them. Similarly, Malaysia requires the inclusion of sugar and fat in nutrient declarations but only requires the declaration of trans and saturated fats if a claim is made.

### Regulatory governance

Legal instruments should ideally detail monitoring processes and enforcement mechanisms available to sanction instances of non-compliance^(30)^. Monitoring and enforcement are dependent on the legal instrument, with voluntary measures being less likely to be enforceable. Of the thirteen countries with legal instruments that included labelling and nutrient declarations, eleven countries listed specific offences or referenced specific sections of their legal instruments when discussing enforcement measures for non-compliance with the legal instrument, but we found substantial variability in the details provided with regard to implementation, monitoring and enforcement. Seven countries detailed information regarding roles, responsibilities and delegated powers as they relate to key implementation actors, such as food analysts, officers, officials and investigators (Table 4).

**Table 4.** Monitoring and enforcement mechanisms in food-related legal instruments of the Western Pacific Region

Mechanisms for monitoring and enforcement	Number of countries
Delegated monitoring authority	
Food analysts/officers/officials/investigators	7
Monitoring system	
Licence/HACCP plan/registration	6
Did not specify measures to monitor	2
Specified an inspection schedule	2
Enforcement mechanisms	
Monetary fine	11
Licence/registration/permit cancellation/suspension	10
Imprisonment	6
Not stated	2
Publication of the names and businesses of offenders	2

Two countries did not specify any measures to monitor compliance with their legal instruments. Two countries included inspection schedules to ensure the compliance of food manufacturers and retailers with legal instruments. For example, Fiji has tightened, normal and reduced inspection schedules for premises, depending on the level of compliance observed in the business.

Six countries required food business operators to hold a licence to operate, a HACCP plan or a business registration, providing a leverage point for enforcing compliance with food labelling regulations. While not all countries had monitoring measures in place, there were a range of enforcement mechanisms to address non-compliance with their legal instruments. Eleven countries specified fines for infringements (ranging from $600 USD in the Solomon Islands to $2·1 million USD in Japan, though it was unclear whether these would apply in the instance of a breach related to nutrient declarations only. There was often little guidance as to how these fines were applied, such as per product or over a period of time. Countries such as the Philippines and the Solomon Islands did include a scale of penalties for first, second and third offences, but it is unclear whether these are applied per product or otherwise.

Imprisonment was also listed as a possible sanction for non-compliance in six countries, with prison sentences ranging from 12 months to 20 years. For example, in Kiribati, the Food Safety Act of 2006 states that any person who labels, sells or advertises food in any way violates the regulations of the Act and is open to a prison sentence of 12 months and/or a fine. The legislation does not specify what the threshold is for the application of these penalties, such as a certain number of products or over a particular period of time.

Ten countries stated licence, registration and permit cancellation or suspension as penalties for non-compliance. For example, in the Philippines, authorisation suspension for the period of 1–6 months was applicable if the provisions of the Act were violated^(34)^. Two countries did not specify any enforcement measures.

## Discussion

This study examined whether legal instruments governing food mandate the declaration of nutrient composition for nutrients of concern in relation to Codex Alimentarius and NCD prevention^(8)^. WHO has underscored the need to adopt food environment policies that reduce overall *demand* for processed HFSS foods, but we found substantial variability in the mandates for nutrient declarations that would enable their implementation and enforcement^(35)^. Thirteen of the twenty-eight countries we reviewed had mandatory nutrient declarations. Only three countries required nutrient declarations that included all the nutrients recommended for display by Codex (energy, protein, available/total carbohydrate, fat, saturated fat, sodium and total sugars), while only four countries required the display of all nutrients recommended by WHO for NCD prevention (fats, saturated fats, trans-fats, sugars and sodium). Several countries required that nutrient declarations be made *only* when the product made a claim in relation to that nutrient.

These findings are significant because nutrient declarations on food labels are foundational to the design, implementation and enforcement of most diet-related NCD prevention policies^(11)^. They also support several case studies from the region, highlighting that insufficient nutrient declarations are a major barrier to the implementation of food and nutrition policies that are critical for diet-related NCD prevention for countries in the WPR^(36–38)^.

This research found that most countries consider legal instruments for food to be a tool for food safety as their primary objective. That none of the legal instruments we reviewed acknowledge the relationship between food standards and NCD prevention as an aim suggests that countries are missing a critical opportunity for food-related legal instruments to act synergistically for both food safety concerns and the group of diseases responsible for the largest share of mortality globally^(1)^. There are emerging examples of where countries have capitalised on this opportunity; for instance, the stated aim of the legal instruments governing food in Uruguay is to ‘identify foods with excessive amounts of specific nutrients’ by introducing declarations for foods high in total fat, saturated fat, sugar or sodium^(32,39)^.

Recognising this challenge, some countries have recently undergone revisions to national food-related legal instruments to expand the set of nutrients being targeted; for instance, in 2017, the Government of Samoa updated its Food (Safety and Quality) Regulations to introduce mandates for sodium declarations as a part of a sodium reduction strategy^(16,37)^. However, in many jurisdictions, the legal instruments for food have been in place for a long period, with four countries only mandating nutrient declarations for particular nutrients ‘where a claim is made’, which is aligned with earlier versions of relevant Codex standards. It is possible that legal instruments are assumed by health policymakers to be providing sufficient coverage. This suggests that a review of food-related legal instruments to ensure labelling mandates are aligned with CODEX guidelines and require the declaration of nutrients of concern, regardless of whether or not a claim is made against them, could be helpful in all settings.

Such diversity in mandatory nutrient declarations has three major implications for policymaking. First, these differences weaken the public health imperative behind BOP nutrient declarations, including the fact that the need for nutrition declarations is evidence-based^(40)^. Second, divergent legal instruments for nutrient declarations make it difficult to adopt a consistent approach to diet-related NCD policymaking across neighbouring countries with a shared food environment context, even where there are intentions to do so^(41)^. Third, this level of discordance across national legal instruments weakens the power of countries to make policies that impact food companies, where together they carry greater influence and buying power^(40,41)^. There is global recognition of the need to improve nutrition legislation through policy harmonisation, including nutrient declarations^(42)^. Standardising nutrient declaration legislation across countries would improve efficiency for manufacturers and government agencies overseeing compliance with non-tariff measures, including food regulations^(43,44)^. It would also improve consumer confidence, providing a fairer playing field commercially^(42)^. Consistency in the minimum nutrient declarations would facilitate greater cooperation across sectors and countries in adopting, implementing and enforcing regulatory measures linked to nutrient contents, building the imperative for coherent regulations across countries with shared food supplies or borders. Coherence in food labelling regulations would be beneficial for companies participating in global markets, who may otherwise have to produce different labels for each country. Regional leaders in the WPR have identified that inconsistency in food labels, including nutrient declarations, has made it difficult to uphold these accountability systems or to enforce the food environment regulations that rely on them^(45,46)^, a challenge that is exacerbated by limited capacity for food and nutrition policy in many settings around the world^(46,47)^. WHO has noted that clear guidelines and shared responsibility for different elements of monitoring, evaluation and enforcement are important for the implementation and feasibility of nutrition labelling^(46)^. Many of the legal instruments reviewed here included enforcement strategies, but these related to the legal instrument in its entirety rather than specific aspects, and food labelling may be considered less acute than food safety breaches.

References to guidelines made by the Codex Alimentarius Commission throughout these legal instruments suggest that Codex plays a critical role in facilitating policy coherence. While Codex standards are in principle voluntary, in practice, they have acted as a ‘floor’ or minimum standard in many areas of food regulation. They are also recognised by agreements of the World Trade Organization as relevant international standards, providing an additional level of legal protection in international trade law for countries that base their national regulations upon them. This makes them critical for providing governments with internationally recognised standards that can be used to hold food producers, retailers and traders accountable and to ensure food safety and quality for both domestic and imported food^(48)^. Ideally, Codex nutrient declaration guidance would more definitively back the need for mandatory declarations of all nutrients recommended by WHO for NCD prevention (SFA, trans-fatty acids, free sugars and sodium). FAO and WHO have a role to play in supporting countries to review the way they regulate nutrient declarations, and strongly encouraging full alignment with Codex guidance could have significant implications for facilitating the uptake of policies to prevent diet-related NCD. It would create space for a stronger and more coherent prevention policy, reducing the burden on countries to continuously defend food labelling measures against industry objections or during trade disputes. This is significant because trade or industry complaints have been a major barrier to the adoption of food environment regulations aimed at reducing diet-related NCD^(48,49)^.

The food industry often opposes stronger nutrient labelling provisions because they are critical for implementing restrictions on the sales and marketing of processed HFSS foods. Updating food labels to accommodate a more comprehensive set of nutrition declarations is likely to have some resourcing implications for companies (e.g., label design, printing and nutrient analysis), though evidence suggests that the resource implications of revising legal instruments for nutrient labelling are outweighed by the health gains that result from implementing such policies^(46)^. There may also be a need to adopt complementary interventions to improve compliance with revised legal measures, for instance, by offering training and technical advisory services to support food manufacturers in meeting new standards.

There is a well-established precedent for introducing or upgrading the scope of nutrient declaration mandates in legal instruments^(32)^. However, more widespread adoption of nutrition declarations will likely require greater support from the technical bodies supporting countries on public health, food and trade matters. For instance, WHO’s forthcoming Guideline on Nutrition Labelling calls for better adherence to the existing Codex standards^(50)^. With so few countries in the WPR mandating nutrient declarations aligned with NCD prevention goals, it would be valuable to expand our search to other WHO regions. While we documented what was stated in the various legal measures for nutrient declarations, further research could evaluate how countries have implemented, monitored and enforced these legal instruments in practice, given that the feasibility of most food environment policies is dependent on the implementation of these labelling declarations. Given the importance of nutrient declarations to food-related prevention policies, this should be prioritised at the global policy level.

### Strengths and limitations

To the best of our knowledge, this is the first systematic review of food labelling regulations against this research question. The use of food-related legal instruments as an existing, publicly available data set was an efficient way to source data across multiple countries. While there can be issues of reliability and validity whenever document analysis is undertaken, we used a systematic method to collect and analyse the data, with two of the co-authors involved in reviewing the policy collection method, data extraction and analysis to mitigate these risks^(27)^. There were three countries for which we could not find or utilise appropriate policy documentation. With few exceptions, food regulations were sourced only at the national level, so we may have missed instances where subnational policy was built on national legislation. This approach was taken because the packaged food supply is shared across states and food labelling laws are predominantly held at the national level.

### Conclusion

Our research found significant variability in legal instruments related to nutrient declarations in the WPR, with very few countries mandating the display of nutrients recommended to be targeted for food policy for NCD prevention. Supporting countries in reviewing legal instruments for nutrient labelling and promoting greater harmonisation would be a relatively straightforward process with significant benefits for strengthening the implementation of food and nutrition policies that are critical for diet-related NCD prevention^(36,37)^.

## Supporting information

Fries et al. supplementary material Fries et al. supplementary material

## Supplementary material

For supplementary material accompanying this paper, visit https://doi.org/10.1017/S1368980025101687
