# Supplementary material for: How food regulations help or hinder the implementation of policies to promote healthy population diets: a review of food regulations in the Western Pacific Region
Source: Public Health Nutr. 2025 Dec 26;29(1):e13. doi: 10.1017/S1368980025101687 (PMC12895442; doi:10.1017/S1368980025101687)
Supplement: Fries et al. supplementary material [file S1368980025101687sup001.docx]

**Appendix A: Search Strategy**

*Platforms searched*

- Google
- Pacific Islands Legal Information Institute (PacLII)
- FAOLEX Database
- Trade Portals

*Search terms*

The following search terms were used in various combinations:

Primary terms:

- “back of pack label”
- “food labelling legislation”
- “food act”
- “food regulations”
- “food code”
- “food safety act”
- “food regulations”
- “labelling of prepackaged foods”
- “labelling standards”
- “nutrition labelling”
- “nutrient label”
- “nutrition labelling guidelines”
- “nutrient labelling policy”
- “nutrition labelling standards”

We combined the search terms above with the names of all 37 countries and areas in the Western Pacific region in our search queries. These countries and areas are:

American Samoa (US), Australia, Brunei Darussalam, Cambodia, China, Cook Islands, Fiji, French Polynesia (France), Guam (US), Hong Kong SAR (China), Japan, Kiribati, Laos, Macao SAR (China), Malaysia, Marshall Islands, Micronesia (Federated States of), Mongolia, Nauru, New Caledonia (France), New Zealand, Niue, Northern Mariana Islands (US), Palau, Papua New Guinea, Philippines, Pitcairn Islands (UK), Republic of Korea, Samoa, Singapore, Solomon Islands, Tokelau, Tonga, Tuvalu, Vanuatu, Vietnam, and Wallis and Futuna (France).

**Appendix B: Legislative Instruments Included in the Study**

| **Country** | **Name of legislation** |
| --- | --- |
| Australia & New Zealand | - Australia New Zealand Food Standards Code |
| Australia | - Food Act 1984 (Victoria) - Food Act 2006 (Queensland) - Food Act 2003 (New South Wales) - Food Regulation 2015 (New South Wales) |
| Brunei | - Public Health (Food) Regulations - S 80/00 CAP. 182, Rg 1 - Public Health (Food) Act – Chapter 182 |
| Cambodia | - Prakas on Standard CS 001-2000 on the Labeling of Food Products - Regulation no. 1045 |
| China | - National Food Safety Standard General Standard for the Labeling of Prepackaged Foods |
| Cook Islands | - Food Regulations 2014 - Food Act (No. 23 of 1992-1993) |
| Fiji | - Food Safety Regulations 2009 - Food Safety Act 2003 |
| Guam | - Guam Food Code - Title 9 of the CFR - Labeling, Marking Devices, and Containers - Title 21 of the CFR - Food and Drugs |
| Hong Kong | - Food and Drugs (Composition and Labelling) Regulations - Cap. 132 sub. leg. W |
| Japan | - Nutrition Labeling Standard - Food Labeling Act – Act No. 70 - Food Sanitation Act – Act No. 233 |
| Kiribati | - Food Regulations and Standards 2014 - Food Safety Act 2006 - Consumer Protection Act 2000 |
| Laos | - Law on Consumer Protection (Unofficial Translation) – No. 02/NA - Order of Minister on Lao Language Labelling (Unofficial Translation) - No. 2501-MOIC.DTD |
| Malaysia | - Food Regulations 1985 - Food Act 1983 – Act 281 |
| Marshall Islands | - Food Safety Act 2010 - 7 MIRC Ch.19 |
| Mongolia | - Technical Regulation on Trading and Distributing of the Certain Imported Prepackaged Goods with Labelling in Mongolian Language in the Domestic Market (Unofficial Translation) - Food Law 1999 |
| Nauru | - Food Safety Act 2005 (Act No. 4) |
| Northern Mariana Islands | - Pure Food, Drug and Cosmetic Device 2 Act of 1998 - H.B. NO. 11-101, HD1 - Public Health Food Regulations |
| Palau | - Republic of Palau Labeling Act - RPPL No. 9-56 2015 |
| Papua New Guinea | - Food Sanitation Regulation 2007 (No. 1 of 2007) - Food Sanitation Act 1991 (No. 29 of 1991) |
| Philippines | - Revised Rules and Regulations Governing the Labeling of Prepackaged Food Products Further Amending Certain Provisions of Administrative Order No. 88-B s. 1984 - Administrative Order No. 2014-0030 - Food Safety Act 2013 – Republic Act No. 10611 |
| Republic of Korea | - Labeling Standards of Foods, Etc. - Notification No. 2019-97 - Act on Labeling and Advertising of Foods - Act No. 18445 |
| Samoa | - Food (Safety and Quality) Regulations 2017 |
| Singapore | - Food Regulations - Sale of Food Act |
| Solomon Islands | - Pure Food (Food Control) Regulations 2010 - Pure Food Act, 1996 |
| Tonga | - Consumer Protection (Product Safety & Labelling Standards) Regulations - Consumer Protection Act |
| Tuvalu | - Food Safety Act |
| Vanuatu | - Food (Control) Regulation Order No. 01 of 2007 - Food Control Act 2006 |
| Vietnam | - No. 43/2017/ND-CP Decree on Goods Labeling |
